# Erenumab for headaches in idiopathic intracranial hypertension: A prospective open‐label evaluation

**DOI:** 10.1111/head.14026

**Published:** 2020-12-14

**Authors:** Andreas Yiangou, James L. Mitchell, Claire Fisher, Julie Edwards, Vivek Vijay, Zerin Alimajstorovic, Olivia Grech, Gareth G. Lavery, Susan P. Mollan, Alexandra J. Sinclair

**Affiliations:** ^1^ Metabolic Neurology Institute of Metabolism and Systems Research College of Medical and Dental Sciences University of Birmingham Birmingham UK; ^2^ Centre for Endocrinology Diabetes and Metabolism Birmingham Health Partners Birmingham UK; ^3^ Department of Neurology University Hospitals Birmingham NHS Foundation Trust Birmingham UK; ^4^ Birmingham Neuro‐Ophthalmology Unit Ophthalmology Department University Hospitals Birmingham NHS Foundation Trust Birmingham UK

**Keywords:** calcitonin gene‐related peptide monoclonal antibody, headache, idiopathic intracranial hypertension, papilledema, raised intracranial pressure

## Abstract

**Objective:**

To determine the effectiveness of erenumab in treating headaches in idiopathic intracranial hypertension (IIH) in whom papilledema had resolved.

**Background:**

Disability in IIH is predominantly driven by debilitating headaches with no evidence for the use of preventative therapies. Headache therapy in IIH is an urgent unmet need.

**Methods:**

A prospective, open‐label study in the United Kingdom was conducted. Adult females with confirmed diagnosis of IIH now in ocular remission (papilledema resolved) with chronic headaches (≥15 days a month) and failure of ≥3 preventative medications received erenumab 4‐weekly (assessments were 3‐monthly). The primary end point was change in monthly moderate/severe headache days (MmsHD) from baseline (30‐day pretreatment period) compared to 12 months.

**Results:**

Fifty‐five patients, mean (SD) age 35.3 (9) years and mean duration of headaches 10.4 (8.4) years with 3.7 (0.9) preventative treatment failures, were enrolled. Mean baseline MmsHD was 16.1 (4.7) and total monthly headache days (MHD) was (29) 2.3. MmsHD reduced substantially at 12 months by mean (SD) [95% CI] 10.8 (4.0) [9.5, 11.9], *p* < 0.001 and MHD reduced by 13.0 (9.5) [10.2, 15.7], *p* < 0.001. Crystal clear days (days without any head pain) increased by 13.1 (9.5) [9.6, 15.3], *p* < 0.001, headache severity (scale 0–10) fell by 1.3 (1.7) [0.9, 1.9], *p* < 0.001, and monthly analgesic days reduced by 4.3 (9.2) [1.6, 6.9], *p* = 0.002. All these measures had improved significantly by 3 months, with a consistent significant response to 12 months. Headache impact test‐6 score and quality of life Short Form‐36 Health Survey significantly improved at 12 months. Sensitivity analysis revealed similar results for patients with and without a prior migraine diagnosis (28/55 (52%) patients) or those with or without medication overuse (27/55 (48%) patients).

**Conclusions:**

This study provides evidence for the effectiveness of erenumab to treat headaches in IIH patients with resolution of papilledema. It provides mechanistic insights suggesting that calcitonin gene‐related peptide is likely a modulator driving headache and a useful therapeutic target.

**Abbreviations**
CGRP: calcitonin gene‐related peptide
HADS: hospital anxiety and depression scale
HIT‐6: headache impact test‐6
ICP: intracranial pressure
IIH: idiopathic intracranial hypertension
MOH: medication overuse headache
MCS: mental component summary score
MHD: monthly total headache days
MmsHD: monthly moderate/severe headache days
NRS: numeric rating scale
OCT: optical coherence tomography
PCS: physical component summary score
Q‐No: four‐item self‐fulfilled questionnaire to predict nocebo effect
SD: standard deviation
SF‐36: Short Form‐36 Health Survey

## INTRODUCTION

Idiopathic intracranial hypertension (IIH) is a chronic disease characterized by raised intracranial pressure (ICP) often associated with younger age, obesity, and females.^1^, ^2^ Disability in IIH is predominantly driven by debilitating headaches that significantly impact quality of life.^3^, ^4^ Reduction in ICP can improve headaches in IIH,^5^, ^6^ but headaches often persist despite normalization of ICP.^4^, ^7^, ^8^, ^9^ Patients with prior IIH with resolved papilledema are termed “IIH in ocular remission” and frequently have a high long‐term headache morbidity.^1^ Headaches in IIH typically have migraine‐like characteristics (approximately 80%).^8^ There is no evidence for the use of headache preventative therapies for IIH headache with limited choices due to the risk of weight gain or mood disorders.^10^

The incidence of IIH is increasing markedly (350% increase in 10 years) with increased economic burden as headaches drive frequent presentations to hospitals.^11^, ^12^ Headache therapy is consequently an urgent unmet need in IIH.^13^

Calcitonin gene‐related peptide (CGRP) is implicated in migraine etiology with elevated levels noted during migraine attacks, levels normalizing after therapeutic triptan administration, and exogenous administration of CGRP precipitating migraine‐like headache in migraineurs.^14^, ^15^, ^16^, ^17^ Monoclonal antibodies targeting the CGRP signaling pathway are efficacious and well‐tolerated therapies for both episodic and chronic migraine and are currently licensed for treatment.^18^, ^19^, ^20^, ^21^, ^22^, ^23^, ^24^ Data are emerging supporting the role of CGRP in posttraumatic headache.^25^ We hypothesized that CGRP would also have a role in driving IIH headaches.

This open‐label prospective evaluation aimed to determine the effectiveness of erenumab in treating headaches in patients with IIH in ocular remission (resolved papilledema).

## METHODS

This study was approved and registered at University Hospitals Birmingham National Health Service Foundation Trust (UHB NHS FT), United Kingdom (Clinical Audit Registration and Management System: CARMS‐15001) with data collection approved by NHS National Research Ethics Committee (14/LO/1208), IIH:LIFE study. The study was conducted from October 2018 to August 2020 with ongoing patient follow‐up in routine care and analysis performed in August 2020. This was a prospective evaluation and we report the primary analysis of the data.

### Study patients

Patients were recruited and data collected prospectively from a tertiary Neuroscience public sector, Headache Centre at UHB NHS FT, UK. Adult patients with IIH in ocular remission and chronic headaches presenting to the Headache Centre were screened for suitability of erenumab therapy (Aimovig^®^, Novartis). Patients were treated with erenumab on a free of charge scheme through the NHS funded by Novartis. The number of patients recruited was determined by the recruitment of every consecutive patient who met the eligibility criteria from October 2018 to August 2019. All patients received free treatment throughout their care.

Eligible patients had chronic moderate/severe headaches (≥15 per month).^26^, ^27^ All patients had IIH in ocular remission, defined as previous diagnosis of IIH with resolution of papilledema.^1^ Study patients were adults (≥18 years) for whom ≥3 prior prophylactic treatments had failed. Treatment failure was defined as any of the following: inadequate efficacy with appropriate dosing and treatment duration; intolerable adverse effects; contraindications preventing use; safety concerns.^28^ Drug treatment attempts were defined as: inadequate efficacy with appropriate dosing and treatment duration or intolerable adverse effects. Patients with a history of headache attributed to intracranial hypotension (apart from short‐lasting post‐lumbar puncture headache) were excluded. Patients were not on topiramate or acetazolamide at erenumab initiation.

### Study design

An open‐label, prospective cohort study was conducted to investigate use of erenumab in chronic headache in patients with IIH in ocular remission. Patients had access to a full range of advice from the Headache Centre as part of routine care. Patients with all degrees of medication overuse headache (MOH), including opiate overuse were included.^28^ MOH was defined according to the International Classification of Headache Disorders 3b: “headache occurring on ≥15 days/month in a patient with a pre‐existing primary headache and developing as a consequence of regular overuse of acute or symptomatic headache medication (≥10 or ≥15 days/month, depending on the medication) for more than 3 months.”^26^

Following the baseline monthly headache diary, patients meeting the eligibility criteria were fully informed of the potential benefits and side effects and signed a written consent to proceed with subcutaneous erenumab (Aimovig^®^, Novartis). A full neurological and ophthalmological review was performed by a neurologist and a neuro‐ophthalmologist including optical coherence tomography (OCT) imaging (Heidelberg Engineering SPECTRALIS, Heidelberg, Germany) and dilated slit‐lamp examination of the fundus to confirm resolution of papilledema.

Patients were taught erenumab self‐administration and commenced at 70 mg 4‐weekly injections. Response was assessed at 3‐monthly follow‐up consultations by phone or clinic visit over a 12‐month period. Monthly headache diaries, IIH symptoms, and Headache Impact Test (HIT‐6)^29^ were reviewed every 3 months; Medical Outcomes Study Short Form (SF‐36) Health Survey (RAND 36‐Item Health Survey 1.0),^30^, ^31^ hospital anxiety and depression scale (HADS),^32^ and four‐item self‐fulfilled questionnaire to predict nocebo effect (Q‐No) questionnaire^33^ were reviewed at baseline, 6 months, and 12 months. Monthly moderate/severe headache days (MmsHD), monthly total headache days (MHD), headache severity (0–10 numerical rating scale [NRS]), crystal clear days (day without any head pain), days using analgesia, and symptoms associated with IIH (but not pathognomonic as they can also be reported in other headache conditions^34^) were derived from the monthly headache diaries.

Dosing was determined from the change in MmsHD, MHD, headache severity, or HIT‐6 score at the 3‐monthly follow‐up consultations. Where the response was ≤50% but ≥30% the dose was increased to 140 mg. Those with ≥50% response continued on erenumab at the 70 mg dose. Those with <30% response were discontinued from treatment.

### Study outcomes

Primary end point was the mean change in MmsHD between the 30‐day pretreatment period compared to 12 months. Data were also reported at 3, 6, and 9 months. A moderate/severe headache day was defined as a day with moderate or severe pain that last at least 4 hours or those requiring the use of abortive therapy.^28^

Secondary end points included the MHD. MHD were all headache days, defined as those with an onset, continuation or recurrence any severity or phenotype of headache, lasting at least 30 minutes.^8^ Other secondary end points were: crystal clear days (days with no headache), change in headache severity (headache severity was evaluated using a NRS; 0 [no pain] to 10 [most severe pain]), monthly analgesic days, responder rates (percentage of patients achieving at least 30%, 50%, and 75% reduction of MmsHD and MHD), and symptoms associated with IIH (double vision, blurred vision, pulsatile tinnitus, and visual obscurations).^1^ Patient reported outcome measures included absenteeism (days off duty due to disability), presenteeism (days with loss of productive capacity during duties),^35^ HIT‐6 at all time‐points, and HADS, SF‐36 (physical component summary score [PCS] and mental component summary score [MCS]), and Q‐No at baseline, 6, and 12 months. Outcomes were in line with the guidelines of the International Headache Society (IHS) for studies of preventive treatment of chronic migraine in adults.^28^ Patient reported outcomes are not specific to IIH (as these have not been yet developed) but have been used in previous IIH trials.^36^, ^37^, ^38^

### Statistical analysis

Statistical analyses were undertaken on SPSS (Armonk, NY: IBM Corp. Version 25.0 [2017]). Data were predominantly normally distributed and consequently represented as mean and standard deviation (SD) unless otherwise specified. End points were compared between the 30‐day pretreatment period and 12 months, with secondary analyses at 3, 6, and 9 months, using a two‐tailed paired *t*‐test where data were normally distributed or Wilcoxon signed rank test where data were not normally distributed. A prespecified subgroup analyses was performed on those with and without MOH at baseline.^28^ Additionally, we analyzed those with and without a preexisting diagnosis of migraine prior to being diagnosed with IIH. We have performed a further post hoc sensitivity analysis for key end points to account for the missing data for patients that erenumab was stopped through imputation by carrying forward the last observed values at the time‐point that erenumab was stopped for the subsequent time‐points. Statistical significance was considered at *p* < 0.05 level (two‐tailed) unless otherwise stated and *p*‐values were unadjusted. As this was a prospective service evaluation study, no power calculations were performed. All patients available for follow‐up were included in the analysis. Missing data were noted in the results and all the authors had access to the study data.

## RESULTS

### Demographics

**FIGURE 1:**
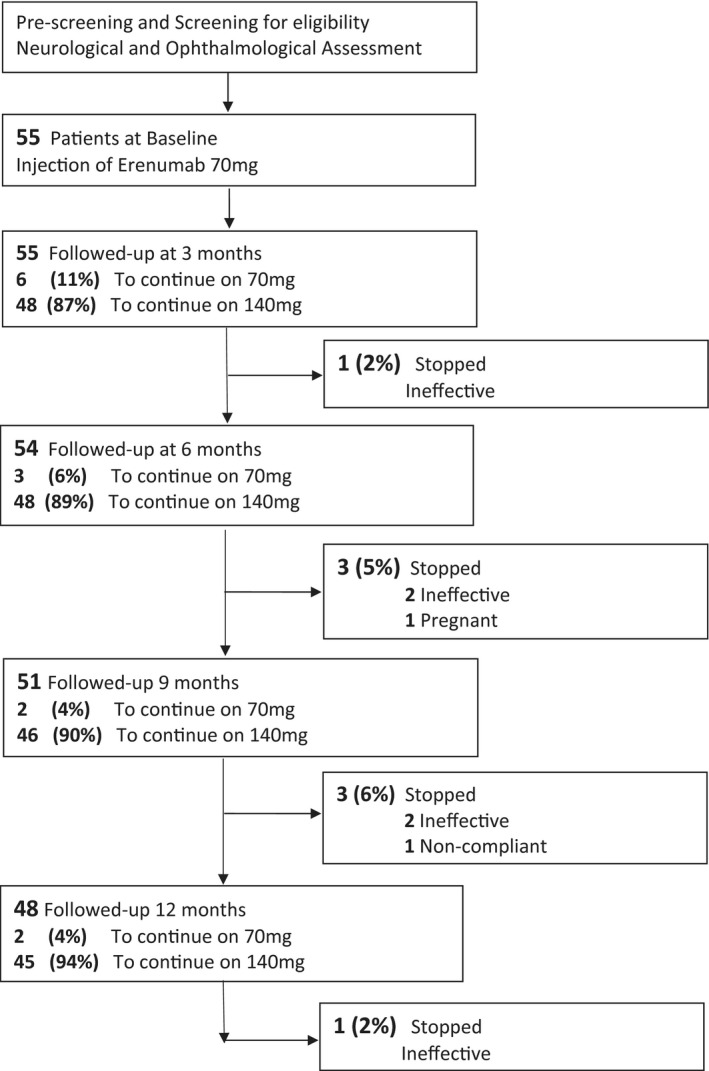
Flowchart of patient recruitment and follow‐up

Fifty‐five patients with IIH in ocular remission and chronic headaches were included in the study (Figure 1). The headache phenotype was chronic migraine‐like in 55/55 (100%) of the patients. All patients were female, with a mean (SD) age of 35.3 (9) years. The duration of chronic headaches since the papilledema had resolved was 1.7 (2.5) years. MOH occurred in 27/55 (48%) and family history of migraine in 24/55 (44%) patients (Table 1).

**TABLE 1:** Baseline patient characteristics

Characteristic	Years, mean (SD)
Total cohort, no. (all female)	55
Age	35.3 (9)
Duration of chronic headache	10.4 (8.4)
Duration of chronic headaches by subgroup	
Migraine prior to IIH	15.6 (8.2), (52%, *n* = 28)
No migraine prior to IIH	5.1 (4.3), (48%, *n* = 27)
Duration of headache following resolution of papilledema (ocular remission)	1.7 (2.5)

History of previous preventative treatment failure	Mean (SD)
Drug treatment trial attempts^a^	2.2 (1.5)
Failed drugs^b^	3.7 (0.9)

	No. (%)
Trial attempt of ≥1	49 (89%)
Trial attempt of ≥2	33 (60%)
Trial attempt of ≥3	20 (36%)
Trial attempt of ≥4	13 (24%)
Failure of ≥3	55 (100%)

Clinical characteristic	No. (%)
Migraine prior to IIH	28 (52%)
No migraine prior to IIH	27 (48%)
Childhood migraine	9 (16%)
Teenage migraine	26 (47%)
Family history of migraine	24 (44%)
Medication overuse headache (MOH)	27 (48%)
Opioid use	16 (29%)
Mental health comorbidities (anxiety/depression)	22 (40%)
Significant alcohol history	0

	Mean (SD), unless otherwise specified
Monthly moderate/severe headache days (MmsHD)	
Baseline	16.1 (4.7)
12 months prior	14.5 (5.2)
Monthly total headache days (MHD)	
Baseline [median (IQR)]	29.0 (2.3) [30 (30–30)]
12 months prior [median (IQR)]	28.3 (3.8) [30 (30–30)]
Headache severity (NRS) baseline	
Baseline	6 (1.3)
12 months prior	6.2 (1.7)
Monthly analgesic days	9 (7.3)
Monthly triptan days	2.9 (4.1)
Monthly crystal clear days [median (IQR)]	0.9 (2.1) [0 (0–0)]
Monthly absenteeism days [median (IQR)]	11.8 (11.5) [7.5 (2–20)]
Monthly presenteeism days [median (IQR)]	21.1 (8) [20 (15–30)]
HADS anxiety score	10.1 (4.6)
HADS depression score	8.9 (4.3)
HIT‐6 score	67.2 (4.4)
Q‐No score	15.3 (6.5)
SF‐36—Physical summary score^c^	36.3 (18)
SF‐36—Mental summary score^c^	38.2 (15.6)

^a^ Drug treatment attempts: ineffective/intolerant.

^b^ Failed drugs: ineffective/intolerant/contraindicated/safety concerns.

^c^ *n* = 53.

Abbreviations: HADS, hospital anxiety and depression scale; HIT‐6, headache impact test‐6; IIH, idiopathic intracranial hypertension; IQR, Interquartile range; MHD, monthly total headache days; MmsHD, monthly moderate/severe headache days; NRS, numeric rating scale (0 = no pain to 10 = worst imaginable pain); Q‐No, four‐item self‐fulfilled questionnaire to predict nocebo effect; SF‐36, Short Form‐36 Health Survey.

The majority of patients 48/55 (87%) required a dose increase at the 3 months, while 6 (11%) continued on 70 mg. At 6 months, 3 (6%) continued on 70 mg and in 48 (89%) the dose was 140 mg (Figure 1). At 9 months, 2 (4%) continued on 70 mg and in 46 (90%) the dose was 140 mg. At 12 months, 2 (4%) continued on 70 mg and in 45 (94%) the dose was 140 mg. By 12 months, erenumab had been discontinued in eight (15%) patients (six ineffective, one pregnant, and one noncompliant).

### Baseline headache, medications, quality of life, and IIH characteristics

The population recruited had severe chronic resistant headache. At baseline the mean duration of headaches was 10.4 (8.4) years. Among the subgroup with migraine prior to IIH, the duration of headaches was 15.6 (8.2) years, and among those with no migraine prior to IIH diagnosis, the total duration of headaches was 5.1 (4.3) years.

The baseline frequency of moderate/severe headaches and total headache days was high (MmsHD 16.1 (4.7), MHD 29.0 (2.3)), and importantly had been high and refractory to treatment for at least a year (12‐month prior MmsHD 14.5 (5.2) and MHD 28.3 (3.8)). Headache severity was 6 (1.3) NRS with severe impact on activities (HIT‐6 score 67.2 (4.4), Table 1). The mean (SD) number of failed preventative treatments was 3.7 (0.9).

Symptoms reported by patients associated with IIH were noted at baseline: 21/55 (38%) reported variable double vision (without evidence of a cranial nerve palsy on extraocular movement examination), 42/55 (76%) blurred vision, 49/55 (89%) pulsatile tinnitus, and 19/55 (35%) transient visual obscurations. Clinical examination confirmed no cranial nerve palsies and slit‐lamp examination confirmed no ongoing papilledema. Mean (SD) peripapillary retinal nerve fiber layer thickness at baseline was 98 (17.8) µm for right eyes and 100 (20.9) µm for left eyes, as quantified by OCT. During the 12‐month follow‐up, seven patients experienced weight gain and recurrence of papilledema, details of these cases are reported separately^39^ but they remain in the overall analysis to reduce bias from loss to follow‐up.

### Efficacy

**FIGURE 2:**
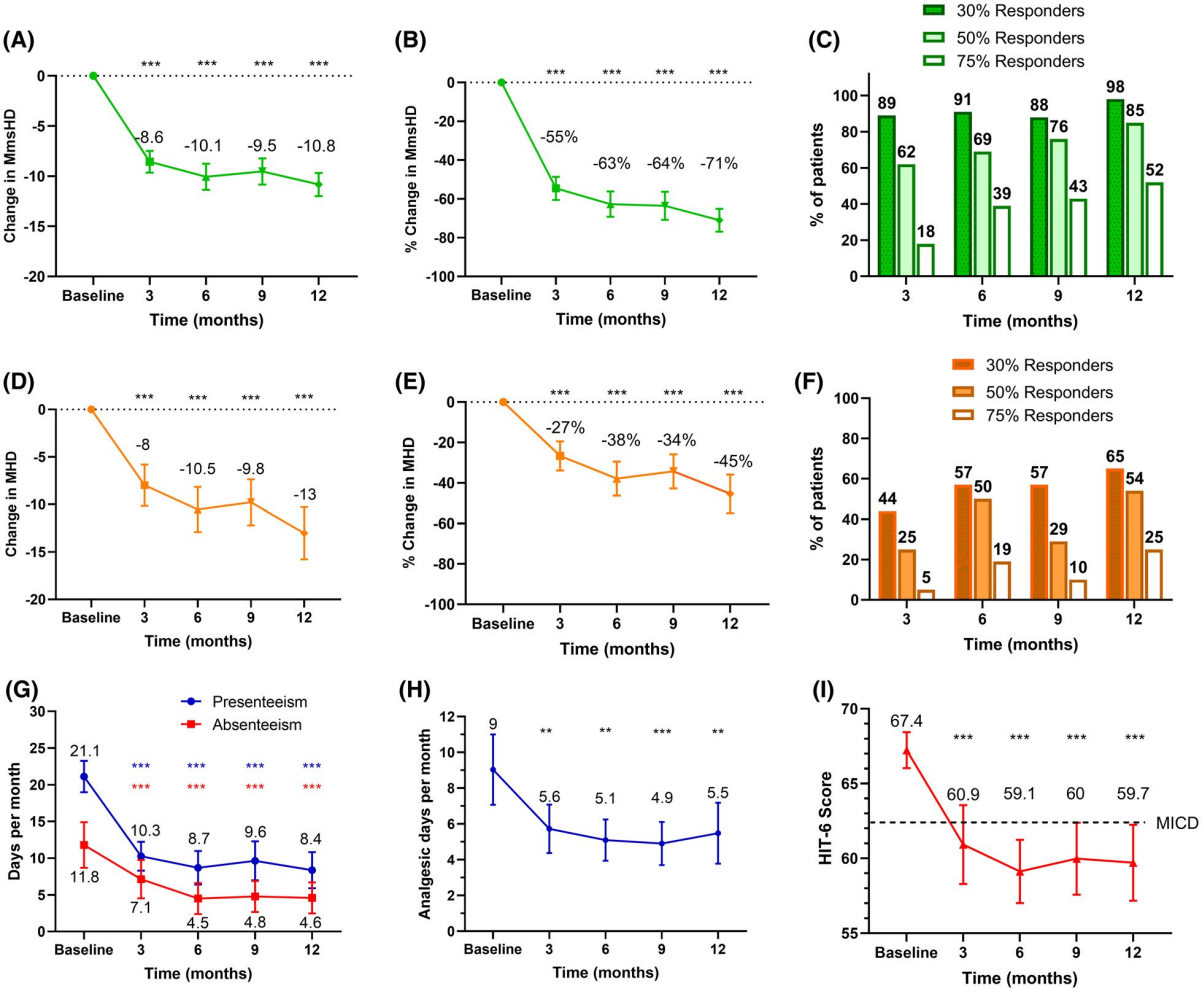
Effect of erenumab on key end points. (A) Change in mean number of monthly moderate/severe headache days from baseline to 3, 6, 9, and 12 months. Error bars represent 95% Confidence intervals (CIs). (B) Percentage change in mean number of monthly moderate/severe headache days from baseline to 3, 6, 9, and 12 months. Error bars represent 95% CIs. (C) Percentage of patients with at least a 30%, 50%, and 75% reduction in their monthly moderate/severe headache days from baseline to 3, 6, 9, and 12 months (responder rates). Number above bars represents the percentage of patients. (D) Change in mean number of monthly total headache days from baseline to 3, 6, 9, and 12 months. Error bars represent 95% CIs. (E) Percentage change in mean number of monthly total headache days from baseline to 3, 6, 9, and 12 months. Error bars represent 95% CIs. (F) Percentage of patients with at least a 30%, 50%, and 75% reduction in their monthly total headache days from baseline to 3, 6, 9, and 12 months (headache responder rates). Number above bars represents the percentage of patients. (G) Mean number of absenteeism and presenteeism days as reported by patients at baseline and at 3, 6, 9, and 12 months. Error bars represent 95% CIs. (H) Mean number of analgesic days at baseline and at 3, 6, 9, and 12 months. Error bars represent 95% CIs. (I) Mean headache impact test‐6 (HIT‐6) score at baseline and at 3, 6, 9, and 12 months. Error bars represent 95% CIs. MHD, monthly total headache days; MICD, minimally important clinical difference; MmsHD, monthly moderate/severe headache days. ***p < 0.001 compared to baseline, **p < 0.01 compared to baseline, *p < 0.05 compared to baseline [Color figure can be viewed at wileyonlinelibrary.com]

There was a substantial improvement in the primary end point of MmsHD at 12 months (reduction of mean (SD) [95% CI (confidence interval)] −10.8 (4.0) [−9.5, −11.9] days, *p* < 0.001) (Figure 2A, Table 2), with significant improvement from 3 months which continued over the study period to 12 months.

**TABLE 2:** Primary and key secondary end point results

	3 months (*n* = 55)	6 months (*n* = 54)	9 months (*n* = 51)	12 months (*n* = 48)
Primary end point (mean (SD)[95% CI])				
MmsHD change	−8.6 (3.9) [−7.6, −9.7]	−10.1 (4.7) [−8.8, −11.4]	−9.5 (4.7) [−8.3, −11.1]	−10.8 (4.0) [−9.5, −11.9]
*p‐value* ^a^	<0.001	<0.001	<0.001	<0.001
Secondary end points (mean (SD) [95% CI], unless otherwise stated)				
MHD change	−8 (8.1) [−5.5, −9.6]	−10.5 (8.7) [−7.9, −12.8]	−9.8 (8.5) [−7.5, −12.3]	−13.0 (9.5) [−10.2, −15.7]
*p‐value* ^b^	<0.001	<0.001	<0.001	<0.001
Crystal clear days change	7.7 (7.7) [5.6, 9.8]	10.3 (9) [7.8, 12.7]	10.3 (8.8) [7.8, 12.8]	13.1 (9.5) [9.6, 15.3]
*p*‐value^b^	<0.001	<0.001	<0.001	<0.001
Headache severity change (NRS)	−1.2 (1.5) [−0.8, −1.61]	−1.3 (1.6) [−0.8, −1.7]	−1 (1.6) [−0.5, −1.4]	−1.3 (1.7) [−0.9, −1.9]
*p*‐value^a^	<0.001	<0.001	<0.001	<0.001
Monthly analgesic days change	−3.4 (7.8) [−1.3, −5.5]	−4.0 (8.1) [−1.8, −6.2]	−4.4 (7.8) [−2.2, −6.6]	−4.3 (9.2) [−1.6, −6.9]
*p*‐value^a^	0.002	0.001	<0.001	0.002
Monthly triptan days change	−0.3 (4.3) [−0.9, 1.5]	−0.8 (3.7) [−0.3, 1.8]	−0.9 (4) [−0.9, 1.8]	−1 (4.6) [−0.4, 2.4]
*p*‐value^a^	0.615	0.183	0.498	0.153
Responder rates moderate/severe headaches/all headache (%)				
≥30%	89%/44%	91%/57%	88%/57%	98%/65%
≥50%	62%/25%	69%/50%	76%/29%	85%/54%
≥75%	18%/5%	39%/19%	43%/10%	52%/25%
Main patient reported outcomes, mean (SD)				
Monthly absenteeism days change	−4.7 (8.3) [−2.1, −6.5]	−7.0 (11.4) [−3.5, −9.6]	−6.7 (11.9) [−3.0, −9.6]	−7.1 (11.5) [−2.9, −9.5]
*p*‐value^b^	<0.001	<0.001	<0.001	<0.001
Monthly presenteeism days change	−10.8 (8.3) [−8.5, −13.1]	−12.3 (11.1) [−9.4, −15.2]	−11.2 (12.2) [−7.8, −14.6]	−12.7 (10.1) [−9.5, −15.5]
*p*‐value^b^	<0.001	<0.001	<0.001	<0.001
HIT‐6 score change	−6.1 (10) [−3.3, −9.0]	−7.7 (8.3) [−5.3, −10.1]	−6.9 (8.8) [−4.6, −9.6]	−7.6 (8.7) [−4.7, −10.0]
*p*‐value^a^	<0.001	<0.001	<0.001	<0.001

IIH symptoms, No. (%)	Baseline (*n* = 55)	3 months (*n* = 55)	6 months (*n* = 54)	9 months (*n* = 51)	12 months (*n* = 48)
Double vision	21 (38%)	22 (40%)	5 (9%)	7 (14%)	12 (25%)
Blurred vision	42 (76%)	38 (69%)	31 (57%)	35 (69%)	31 (65%)
Pulsatile tinnitus	49 (89%)	47 (85%)	35 (65%)	39 (77%)	35 (73%)
Visual obscurations	19 (35%)	22 (40%)	6 (11%)	7 (14%)	4 (8%)

^a^ Paired *t*‐test compared to baseline.

^b^ Wilcoxon signed ranks test compared to baseline.

Abbreviations: CI, confidence interval; HIT‐6, headache impact test‐6; IIH, idiopathic intracranial hypertension; MHD, monthly total headache days; MmsHD, monthly moderate/severe headache days; NRS, numeric rating scale (0 = no pain to 10 = worst imaginable pain).

The overall MHD also significantly improved from 3 months and at all time‐points to 12 months (MHD reduced by −13.0 (9.5) [−10.2, −15.7] at 12 months, *p* < 0.001) (Figure 2D, Table 2). The responder rates for moderate/severe headache and totalheadache days are shown in Figure 2C,F and notably 85% (41/48) of patients achieved a 50% reduction of moderate/severe headache days and 54% (26/55) achieved a 50% reduction in totalheadache days (Table 2). By 12 months, 52% (25/48) had a ≥75% moderate/severe headache responder rate. The number of crystal clear days increased from 0.9 (2.1) to 8.6 (8.3) at 3 months, 11.2 (9.1) at 6 months, 11.3 (9) at 9 months, and 13.9 (9.8) at 12 months, *p* < 0.001 (Table 2). Headache severity reduced by −1.3 (1.7) [−0.9, −1.9] points at 12 months, *p* < 0.001.

Accompanying the improvements in headaches we noted a consistent significant reduction in frequency of analgesic use from the 3‐month time‐point continuing until 12 months (3 months −3.4 (7.8) [−1.3, −5.5] days, 6 months −4.0 (8.1) [−1.8, −6.2] days, 9 months −4.4 (7.8) [−2.2, −6.6] days, and 12 months −4.3 (9.2) [−1.6, −6.9], *p* = 0.002 (Table 2, Figure 2H). Presenteeism and absenteeism significantly improved (Table 2, Figure 2G).

### Patient reported outcome measures

There was a substantial reduction in the headache disability as reported by the HIT‐6 score (from 67.4 (4.4) at baseline to 60.9 (9.2) at 3 months, 59.1 (7.4) at 6 months, 60 (8.4) at 9 months, and 59.7 (8.6) at 12 months *p* < 0.001 (Table 2, Figure 2I). There were no notable reductions in the HADS‐A, HADS‐D, and Q‐NO score (Table 3). However, quality of life, evaluated at 6 months and 12 months using the SF‐36 score showed significant improvement in the PCS (36.3 (18) at baseline to 44.9 (22.2) at 6 months and 49.0 (24.8) at 12 months) and in the MCS (38.2 (15.6) at baseline to 48.0 (18.1) at 6 months and 45.2 (21.5) at 12 months) (Table 3).

**TABLE 3:** Patient reported outcomes^a^

Mean (SD) [95% CI]	Baseline	6 months	Change	*p*‐value	12 months	Change	*p*‐value
HADS	*n* = 55	*n* = 35			*n* = 31		
Anxiety score	10.1 (4.6)	8.5 (4.8)	−1.3 (4.4) [−0.2, 2.9]	0.083	10.1 (4.9)	−0.3 (3.3) [−0.9, 1.5]	0.593
Depression score	8.9 (4.3)	7.8 (4.7)	−1.1 (4.6) [−0.5, 2.7]	0.163	8.9 (5.5)	0.0 (4.9) [−1.8, 1.8]	1
Q‐No	*n* = 55	*n* = 39			*n* = 28		
Score	15.3 (6.5)	14.1 (5.0)	−1.5 (8.1) [−1.3, 3.9]	0.304	15.0 (4.3)	−1.8 (8.1) [−1.4, 4.9]	0.255
SF‐36	*n* = 53	*n* = 24			*n* = 30		
Physical component summary (PCS)	36.3 (18.0)	44.9 (22.2)	11.7 (21.6) [2.6, 20.8]	0.014	49.0 (24.8)	10.6 (25.4) [1.2, 20.1]	0.029
Mental component summary (MCS)	38.2 (15.6)	48.0 (18.1)	12.6 (17.0) [5.4, 19.8]	0.001	45.2 (21.5)	7.7 (20.0) [0.3, 15.2]	0.043

Note

Data presented as Mean (SD). *p*‐values represent 6 and 12 months change compared to baseline (Paired *t*‐test).

^a^ Only fully completed returned questionnaires were included in the analysis.

Abbreviations: HADS, hospital anxiety and depression scale; Q‐No, four‐item self‐fulfilled questionnaire to predict nocebo effect; SF‐36, Short Form‐36 Health Survey.

### Subanalyses

**FIGURE 3:**
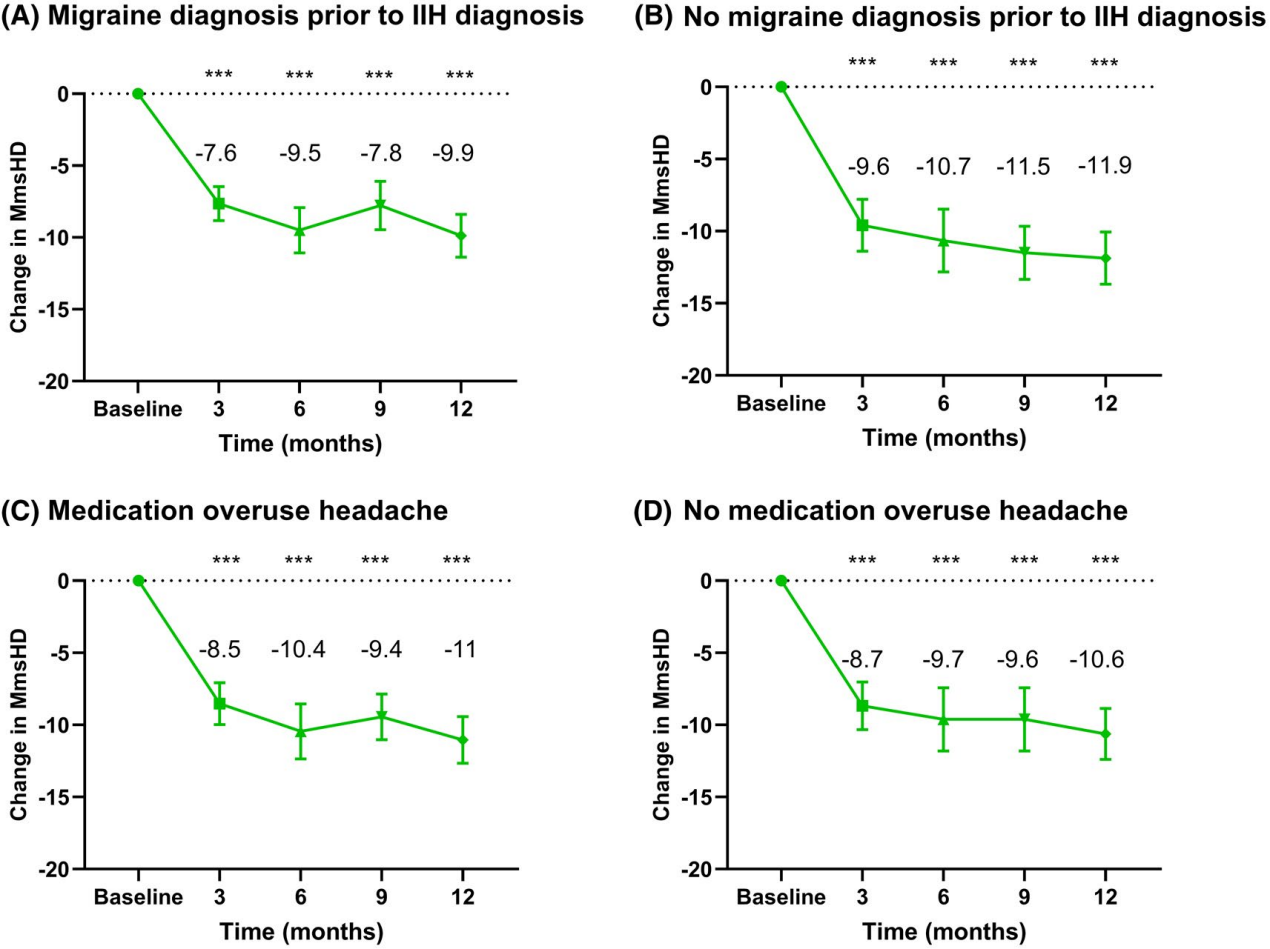
Effect of erenumab on headache in those with and without previous migraine and medication overuse headache. Change in mean number of monthly moderate/severe headache days from baseline to 3, 6, 9, and 12 months. Error bars represent 95% confidence intervals (CIs). (A) Patients with migraine diagnosis prior to IIH diagnosis. (B) Patients without migraine diagnosis prior to IIH diagnosis. (C) Patients with MOH at baseline. (D) Patients without MOH at baseline. IIH, idiopathic intracranial hypertension; MOH, medication overuse headache. ***p < 0.001 compared to baseline [Color figure can be viewed at wileyonlinelibrary.com]

A subanalysis was performed in those with migraine prior to their IIH diagnosis (28/55 (52%), mean (SD) baseline MmsHD 15.3 (4.3)), and those with no prior migraine before their IIH diagnosis (27/55 (48%), mean (SD) baseline MmsHD 16.9 (4.9)). Prior migraine diagnosis was established from review of medical records and patient reporting the diagnosis. Results were also equivalent between these groups with reduction in MmsHD at 3 months −7.6 (3.1) [−6.6, −9.0] and −9.6 (4.5) [−7.8, −11.4], at 6 months −9.5 (4.1) [−8.0, −11.1] and −10.7 (5.4) [−8.5, −12.8], at 9 months −7.8 (4.2) [−6.2, −9.8] and −11.5 (4.4) [−9.7, −13.5], and at 12 months −9.9 (3.6) [−8.2, −11.2], *p* < 0.001 and −11.9 (4.2) [−10.0, −13.8], *p* < 0.001 (Figure 3A,B, Table 4).

**TABLE 4:** Subanalysis of patients with/without migraine prior to IIH diagnosis and with/without MOH

Mean (SD) [95% CI], unless otherwise specified	Baseline	3 months	6 months	9 months	12 months
Patients with migraine diagnosis prior to IIH	*n* = 28	*n* = 28	*n* = 28	*n* = 27	*n* = 25
MmsHD	15.3 (4.3)	7.5 (4.1)	5.7 (4.4)	7 (6.1)	5.0 (4.1)
MmsHD change		−7.6 (3.1) [−6.6, −9.0]	−9.5 (4.1) [−8.0, −11.1]	−7.8 (4.2) [−6.2, −9.8]	−9.9 (3.6) [−8.2, −11.2]
*p*‐value		<0.001	<0.001	<0.001	<0.001
MHD	29.6 (1.1)	22.5 (8.0)	19.6 (8.7)	20.6 (9.2)	16.6 (9.7)
MHD change		−7.1 (7.6) [−4.1, −10.0]	−10.0 (8.6) [−6.7, −13.3]	−8.5 (9) [−5.5, −12.6]	−13.0 (9.7) [−8.4, −16.3]
*p*‐value		<0.001	<0.001	0.001	<0.001
Patients without migraine diagnosis prior to IIH	*n* = 27	*n* = 27	*n* = 26	*n* = 24	*n* = 23
MmsHD	16.9 (4.9)	7.3 (3.8)	6.3 (4.6)	5.6 (5.3)	4.4 (3.8)
MmsHD change		−9.6 (4.5) [−7.8, −11.4]	−10.7 (5.4) [−8.5, −12.8]	−11.5 (4.4) [−9.7, −13.5]	−11.9 (4.2) [−10.0, −13.8]
*p*‐value		<0.001	<0.001	<0.001	<0.001
MHD	28.3 (−2.9)	20.3 (8.5)	17.5 (9.6)	17.9 (9.3)	15.3 (10.0)
MHD change		−8.9 (8.5) [−5.0, −11.0]	−11.1 (8.9) [−7.0, −14.5]	−10.7 (9.3) [−7.4, −14.2]	−13.1 (9.4) [−9.6, −17.8]
*p*‐value		<0.001	0.001	0.001	<0.001
Patients with MOH	*n* = 27	*n* = 27	*n* = 25	*n* = 25	*n* = 24
MmsHD	16.4 (4.7)	7.8 (3.7)	6.0 (4.3)	6.2 (5)	4.5 (3.4)
MmsHD change		−8.5 (3.7) [−7.2, −10.1]	−10.4 (4.8) [−8.6, −12.4]	−9.4 (3.8) [−7.9, −11.3]	−11.0 (3.8) [−9.1, −12.6]
*p*‐value		<0.001	<0.001	<0.001	<0.001
MHD	28.3 (2.9)	20.4 (8.5)	18.8 (9.2)	19.6 (9.7)	16.4 (10.5)
MHD change		−7.6 (7.5) [−4.3, −10.2]	−9.9 (8.7) [−5.9, −13.1]	−8.7 (9.3) [−4.7, −12.3]	−12.0 (10.0) [−7.6, −16.0]
*p*‐value		<0.001	<0.001	0.002	<0.001
Patients without MOH	*n* = 28	*n* = 28	*n* = 27	*n* = 26	*n* = 24
MmsHD	15.7 (4.7)	7.0 (4.2)	6 (4.7)	5.9 (5.6)	5.0 (4.4)
MmsHD change		−8.7 (4.3) [−7.0, −10.3]	−9.7 (4.7) [−7.8, −11.5]	−9.6 (5.4) [−7.6, −12.0]	−10.6 (4.2) [−8.9, −12.4]
*p*‐value		<0.001	<0.001	<0.001	<0.001
MHD	29.6 (1.1)	22.4 (8.1)	18.4 (9.1)	18.5 (8)	15.5 (9.3)
MHD change		−8.3 (8.7) [−4.3, −10.2]	−11.2 (8.8) [−7.7, −14.7]	−11.2 (7.8) [−8.0, −14.3]	−14.1 (9.0) [−10.3, −17.9]
*p*‐value		<0.001	<0.001	0.002	<0.001
Patients without prior migraine and without MOH	*n* = 14	*n* = 14	*n* = 13	*n* = 12	*n* = 12
MmsHD	17.1 (5.2)	6.8 (4.7)	6.2 (5.1)	4.8 (4.2)	5.3 (4.9)
MmsHD change		−10.3 (5.0) [−7.4, −13.2]	−10.9 (5.9) [−7.3, −14.5]	−12.1 (4.8) [−9.5, −15.5]	−12.1 (4.6) [−9.2, −15.0]
*p*‐value		<0.001	<0.001	<0.001	<0.001
MHD	29.6 (0.7)	21.2 (8.8)	16.2 (9.2)	16.9 (6.9)	16.8 (10.6)
MHD change		−10.6 (10.0) [−3.4, −13.4]	−13.5 (9.0) [−8.0, −19.0]	−12.2 (6.8) [−8.5, −17.2]	−12.9 (10.5) [−6.3, −19.6]
*p*‐value		0.008	0.003	0.005	0.008

Note

Data presented as Mean (SD) [95% Confidence Interval], unless otherwise specified. Statistical tests represent paired *t* test for MmsHD and Wilcoxon signed ranks test for MHD for the 3, 6, 9, and 12 months end points compared to baseline.

Abbreviations: CI, Confidence interval; IIH, idiopathic intracranial hypertension; MHD, monthly total headache days; MmsHD, monthly moderate/severe headache days; MOH, medication overuse headache.

A further subanalysis was performed for outcomes in those with MOH (27/55 (48%) mean (SD) baseline MmsHD 16.4 (4.7)) and those without MOH (28/55 (52%) mean (SD) baseline MmsHD 15.7 (4.7)) at baseline. Results were equivalent between groups with reduction in MmsHD at 3 months −8.5 (3.7) [−7.2, −10.1] and −8.7 (4.3) [−7.0, −10.3], at 6 months −10.4 (4.8) [−8.6, −12.4] and −9.7 (4.7) [−7.8, −11.5], at 9 months −9.4 (3.8) [−7.9, −11.3] and −9.6 (5.4) [−7.6, −12.0], and at 12 months −11.0 (3.8) [−9.1, −12.6], *p* < 0.001 and −10.6 (4.2) [−8.9, −12.4], *p* < 0.001 (Figure 3C,D).

Additionally, 14 patients did not have a migraine diagnosis prior to IIH and no MOH and improvements were equivalent with the other groups (Table 4). The above subanalyses were performed for MHD with significant improvements at all time‐points in all subgroups (Table 4).

A sensitivity analysis was conducted to account for missing data (*n* = 7 at 12 months) (last observation carried forward), the change in MmsHD was −10.1 (4.8) [−8.8, −11.4] and MHD −11.4 (9.9) [−8.7, −14.1] at 12 months (*p* < 0.001) (Table 5).

**TABLE 5:** Subanalysis for key end points with missing data imputed

	3 months (*n* = 55)	6 months (*n* = 55)	9 months (*n* = 55)	12 months (*n* = 55)
Primary end point (mean (SD) [95% CI])				
MmsHD change	−8.6 (3.9) [−7.6, −9.7]	−9.9 (4.8) [−8.6, −11.2]	−9.5 (5.1) [−8.1, −10.9]	−10.1 (4.8) [−8.8, −11.4]
*p*‐value	<0.001	<0.001	<0.001	<0.001
Secondary end point (mean (SD) [95% CI])				
MHD change	−8 (8.1) [−5.5, −9.6]	−10.2 (8.9) [−7.8, −12.6]	−9.3 (8.5) [−7.0, −11.6]	−11.4 (9.9) [−8.7, −14.1]
*p*‐value	<0.001	<0.001	<0.001	<0.001

Note

Imputation for missing data performed by carrying forward the last observed values at the time‐point that erenumab was stopped for the relevant patients, for all the subsequent time‐points for those patients. Statistical tests represent paired *t*‐test for MmsHD and Wilcoxon signed ranks test for MHD for the 3, 6, 9, and 12 months end points compared to baseline.

Abbreviations: CI, Confidence interval; MHD, monthly total headache days; MmsHD, monthly moderate/severe headache days.

### Safety and tolerability

Side effects were reported in 21/55 (38%) patients. These included constipation 9 (16%), generalized muscle cramps or spasms 6 (11%), generalized itching 3 (5%), generalized skin rash 3 (5%), injection site pain 3 (5%), hair thinning 2 (4%), nasopharyngitis 1 (2%), and acne 1 (2%). These side effects were not deemed serious and no patient discontinued treatment due to side effects. At 1 year 10/48 (21%) patients reported side effects. Seven patients from our cohort suffered recurrence of raised ICP, as evidenced by a return of the papilledema, however, the headaches did not recur while treated with erenumab. We have reported these patients individually as a case series.^39^

## DISCUSSION

This prospective open‐label study demonstrates the efficacy of erenumab to improve headaches in patients with IIH in whom the papilledema has resolved (ocular remission). The response was marked given that the population recruited had chronic, long duration, resistant headaches, and approximately half had MOH.

In these IIH patients with persistent headaches following resolution of papilledema, the moderate/severe headache days reflected migraine‐like headache days. Erenumab reduced the frequency of moderate/severe headache days by 71% and total headache days by 45% from baseline to 12 months. Further, erenumab significantly increased crystal clear days, reduced analgesic days, reduced severity and reduced absenteeism and presenteeism. Headache disability and quality of life (HIT‐6 score and SF‐36 PCS and SF‐36 MCS) also significantly improved. The majority of patients required an increased dose of erenumab to 140 mg monthly (87% at 3 months, 89% at 6 months, 90% at 9 months, and 94% by 12 months). The treatment was well tolerated with no patient withdrawals related to adverse effects and only six discontinuing due to lack of effect (by 12 months). Similar efficacy was seen in those without a diagnosis of previous migraine (48% of the cohort).

There are few pharmacotherapies in IIH although novel therapeutic approaches are being developed to target reduction in ICP.^36^, ^40^, ^41^, ^42^ However, despite overwhelming evidence of the headache burden in IIH,^4^, ^8^ treatments specifically aimed at managing headache in IIH have not been previously evaluated. In routine care, existing migraine preventative drugs are used off label, without evidence of efficacy in this population. Furthermore, they are often not tolerated or contraindicated in IIH patients due to the risk of exacerbating obesity (a key driver for raised ICP) and mood disorders. Hence, this study marks an important step forward to establish targeted headache treatments for IIH patients with chronic headaches in whom papilledema has resolved.

Our results, while open‐label, are in line with randomized controlled trial data evaluating erenumab in chronic migraine patients.^22^, ^43^ IIH patients with MOH were included, reflecting real‐life populations^44^ and in line with IHS guidelines for chronic migraine trials.^28^ MOH was common among this IIH cohort (48%) as has been noted in previous IIH populations.^8^ This study demonstrates equivalent efficacy of erenumab to reduce headache in those with and without MOH at baseline. Additionally analgesic use reduced significantly over the study period, an outcome also noted in the erenumab chronic migraine trial.^22^

The IIH patients had severely disabling headaches at baseline, as demonstrated by the HIT‐6 score.^45^ The reductions in the HIT‐6 score by −7.6 at 12 months is both clinically and statistically significant (minimal important clinical difference in HIT‐6 is 5).^45^ The improvements in symptoms associated with IIH were only modest, with a fluctuating course through the duration of the study. These symptoms are not pathognomonic for IIH and often associated with other headache disorders and should be interpreted with caution.^34^

Quality of life evaluated by the SF‐36 domains (physical component and MCSs) improved significantly at 12 months (change in PCS 12.2; MCS 8.7, respectively). There was minimal change in the Hospital Anxiety and depression score (HADS) in our study, however, 40% of our cohort had a concurrent mental health disorder being actively managed which may have impacted the results. The Q‐No score was elevated at baseline (>15) reflecting a heightened nocebo response potentially driven by problematic side effects encountered during previous medication trials.^33^ The score did not change greatly during the study (15.3 (6.5) at baseline, decreasing to 15 (4.3) at 12 months).

There was a strong family history of migraine (24/55, 44%) among the IIH patients with resistant chronic headaches recruited into this study. We speculate that, as is noted among chronic posttraumatic headache patients, a family history of migraine may represent a poor prognostic marker for progression of chronic IIH headaches.^46^, ^47^, ^48^

This study suggests that the CGRP pathway is likely a key modulator of headache in patients with IIH and that targeting CGRP may be a useful therapeutic strategy to manage headache. We hypothesize, as is suggested in other secondary headache disorders, that raised ICP drives trigeminovascular activation and CGRP release which continues even after the original insult of the raised ICP has subsided.^25^, ^49^, ^50^ In support of the role of CGRP in driving headache mechanisms in the setting of raised ICP we evaluated the treatment response in IIH patients with and without a history of migraine headaches prior to the diagnosis of IIH. We noted a significant treatment response even in those with no prior migraine diagnosis, suggesting a role of CGRP in headache precipitated by raised ICP.

The open‐label, prospective, study design is limited as there is no comparator group, thus, no ability to assess any potential placebo response which is known to be high with injectable headache therapies.^51^ The placebo response in a randomized controlled trial of erenumab was 23% at 12 weeks.^22^ However, the relatively long (12 months) duration of follow‐up with sustained therapeutic response points away from a predominant placebo response as this would typically wain with time. Open‐labeled studies do need to be interpreted with caution as response may reflect regression to the mean, spontaneous fluctuation, or disease progressing into remission. Furthermore, weight loss can improve headaches in IIH.^6^ During this study, patients were not on a formal weight management program; however, we anticipate that all would have been aware of the importance of weight management in maintaining their IIH in ocular remission. As the majority of follow‐up visits were by phone, weight was not formally assessed, but in those describing weight gain we formally assessed for recurrence of papilledema.^39^ We cannot exclude the possibility that weight loss could have occurred in these patients during follow‐up. However, the above are unlikely to be the principal factors explaining our results as the cohort at baseline were experiencing a consistently high headache load, with similar MmsHD and MHD 12 months prior to baseline (MmsHD 12 months pre‐enrolment 14.5 (5.2) and 16.1 (4.7) at baseline and MHD 12 months pre‐enrolment 28.3 (3.8) and 29 (2.3) at baseline). Thus, the baseline headache frequency at study entry was consistently high and unchanging over a prolonged period (when similar approaches and fluctuations would have occurred) until the erenumab was commenced. Furthermore, a limitation of this study was that non‐responders were not followed up and this could have inflated the outcomes. However very low numbers of patients were lost to follow‐up by 12 months (five ineffective, one pregnant, and one noncompliant). The sensitivity analysis revealed similar significant improvements for key end points even when missing data were imputed (Table 5). Missing data for the primary end point were *n* = 7 but was higher for the patient reported outcome questionnaires as the majority of follow‐up visits were by phone, enabling the majority of the data to be collected at the time of the visit, however, we required patients to post back the questionnaires which led to greater missing data.

## CONCLUSION

This prospective open‐label study of erenumab in IIH patients with persistent headaches in whom their papilledema has resolved, demonstrates significant efficacy to reduce headaches. The moderate/severe headaches days reflected migraine‐like headaches in this IIH cohort. Erenumab had similar efficacy among the 48% who had no prior history of migraine. Targeting CGRP is likely to be a useful therapeutic strategy and worthy of evaluation in a future randomized controlled trial. The mechanisms driving headache in patients with IIH have not been previously evaluated or understood. These data provide important mechanistic insights suggesting that CGRP is likely a key modulator driving headache in patients with IIH.

## ETHICAL APPROVAL

This study was approved and registered at University Hospitals Birmingham National Health Service Foundation Trust, United Kingdom (Registered Code, Clinical Audit Registration and Management System: CARMS‐15001) with data collection approved by NHS National Research Ethics Committee (14/LO/1208), IIH:LIFE study.

## CONFLICT OF INTEREST

Yiangou, Mitchell, Fisher, Vijay, Alimajstorovic, Lavery declare no conflict. Edwards has received speaker fees and Honoria from Novartis, Teva, Eli Lily, and Allergan on headache treatments but not related to IIH. Mollan has received Honoria from Novartis for speaking on topics unrelated to this drug, but within a National headache network meeting (November 2019). Sinclair has received speaker fees and Honoraria from Novartis (erenumab) and Allergan (BOTOX), in addition, Invex therapeutics, company director with salary and stock options (2019, 2020). Grech has received fees for consultancy work for Invex therapeutics (2020). The authors declare no other financial relationships with any organizations that might have an interest in the submitted work; and no other relationships or activities that could appear to have influenced the submitted work.

## AUTHOR CONTRIBUTIONS

*Study concept and design*: Andreas Yiangou, James L. Mitchell, Gareth G. Lavery, Susan P. Mollan, and Alexandra J. Sinclair. *Acquisition of data*: Andreas Yiangou, James L. Mitchell, Claire Fisher, Julie Edwards, Vivek Vijay, Susan P. Mollan, and Alexandra J. Sinclair. *Analysis and interpretation of data*: Andreas Yiangou, James L. Mitchell, Zerin Alimajstorovic, Olivia Grech, Gareth G. Lavery, Susan P. Mollan, and Alexandra J. Sinclair. *Drafting of the manuscript*: Andreas Yiangou, and Alexandra J. Sinclair. *Revising it for intellectual content*: Andreas Yiangou, James L. Mitchell, Claire Fisher, Julie Edwards, Vivek Vijay, Zerin Alimajstorovic, Olivia Grech, Gareth G. Lavery, Susan P. Mollan, and Alexandra J. Sinclair. *Final approval of the completed manuscript*: Andreas Yiangou, James L. Mitchell, Claire Fisher, Julie Edwards, Vivek Vijay, Zerin Alimajstorovic, Olivia Grech, Gareth G. Lavery, Susan P. Mollan, and Alexandra J. Sinclair.

## Data Availability

All data are available from the corresponding author on reasonable request.
